# Schedule-dependent cytotoxicity of SN-38 in p53 wild-type and mutant colon adenocarcinoma cell lines

**DOI:** 10.1038/sj.bjc.6694370

**Published:** 1999-12

**Authors:** R H te Poele, S P Joel

**Affiliations:** Department of Medical Oncology, Barry Reed Oncology Laboratory, Saint Bartholomew’s Hospital, 4th Floor 38 Little Britain, London ECIA 7BE, UK

**Keywords:** SN-38, apoptosis, p53, cell cycle, schedule, colon adenocarcinoma cell lines

## Abstract

In this study the effects of SN-38 on colon adenocarcinoma cell lines expressing wild-type p53 (LS174T) or mutant non-functional p53 (HT29) have been investigated. On exposure to SN-38, HT29 cells rapidly progressed through G1 and S and arrested in G2/M. Release and concomitant increase in apoptosis after 48 h was concentration- and time-dependent (*P* < 0.001), being more rapid at higher concentrations, but reaching plateau at 10 ng ml^–1^ with prolonged exposure. LS174T cells showed only a small increase in apoptosis, and only at high concentrations (50–100 ng ml^–1^). The main effect of SN-38 in LS174T cells was prolonged cell cycle arrest, which was independent of concentration. Arrest occurred in all phases of the cell cycle, with the distribution depending on concentration (*P* < 0.001) and not duration (*P* > 0.05). With increasing concentration, LS174T cells arrested in G2/M, S and G1. Cell cycle arrest was coincident with increased p53 expression in each phase of the cell cycle. Expression in G1 increased with time and concentration (*P* < 0.001, *P* = 0.01 respectively), whereas in S and G2/M p53 expression increased only with time (*P* < 0.001). Dose-dependent p53-associated G1 arrest, in the absence of DNA synthesis indicates an additional cytotoxic mechanism for SN-38, which requires higher concentrations than the S phase mechanism, and detection of which seems to involve p53. For incubations with the same ED (exposure × duration), apoptosis in HT29 cells was significantly higher for prolonged exposure to lower concentrations, whereas in LS174T cells there was a trend towards increased apoptosis with shorter exposures to higher concentrations, indicating a schedule effect of SN-38. Although expression of wild-type p53 leads to a more rapid induction of apoptosis, SN-38 cytotoxicity was generally greater in cells with mutant p53, as wild-type cells escaped apoptosis by p53 associated prolonged cell cycle arrest. Thus, pulsed schedules with higher doses may be more effective in cells expressing wild-type p53, whereas continued exposure with protracted schedules may be more active in cells expressing mutant p53. © 1999 Cancer Research Campaign


*British Journal of Cancer* (1999) **81**(8), 1285–1293
© 1999 Cancer Research Campaign
Article no. bjoc.1999.6843

### Schedule-dependent cytotoxicity of SN-38 in p53 wild-type and mutant colon adenocarcinoma cell lines

**RH te Poele and SP Joel**

Barry Reed Oncology Laboratory, Department of Medical Oncology, 4th Floor 38 Little Britain, Saint Bartholomew's Hospital, London ECIA 7BE, UK

**Summary** In this study the effects of SN-38 on colon adenocarcinoma cell lines expressing wild-type p53 (LS174T) or mutant non-functional p53 (HT29) have been investigated. On exposure to SN-38, HT29 cells rapidly progressed through G1 and S and arrested in G2/M. Release and concomitant increase in apoptosis after 48 h was concentration- and time-dependent ($P$ < 0.001), being more rapid at higher concentrations, but reaching plateau at 10 ng ml$^{-1}$ with prolonged exposure. LS174T cells showed only a small increase in apoptosis, and only at high concentrations (50–100 ng ml$^{-1}$). The main effect of SN-38 in LS174T cells was prolonged cell cycle arrest, which was independent of concentration. Arrest occurred in all phases of the cell cycle, with the distribution depending on concentration ($P$ < 0.001) and not duration ($P$ > 0.05). With increasing concentration, LS174T cells arrested in G2/M, S and G1. Cell cycle arrest was coincident with increased p53 expression in each phase of the cell cycle. Expression in G1 increased with time and concentration ($P$ < 0.001, $P$ = 0.01 respectively), whereas in S and G2/M p53 expression increased only with time ($P$ < 0.001). Dose-dependent p53-associated G1 arrest, in the absence of DNA synthesis indicates an additional cytotoxic mechanism for SN-38, which requires higher concentrations than the S phase mechanism, and detection of which seems to involve p53. For incubations with the same ED (exposure × duration), apoptosis in HT29 cells was significantly higher for prolonged exposure to lower concentrations, whereas in LS174T cells there was a trend towards increased apoptosis with shorter exposures to higher concentrations, indicating a schedule effect of SN-38. Although expression of wild-type p53 leads to a more rapid induction of apoptosis, SN-38 cytotoxicity was generally greater in cells with mutant p53, as wild-type cells escaped apoptosis by p53 associated prolonged cell cycle arrest. Thus, pulsed schedules with higher doses may be more effective in cells expressing wild-type p53, whereas continued exposure with protracted schedules may be more active in cells expressing mutant p53. © 1999 Cancer Research Campaign

**Keywords**: SN-38, apoptosis; p53; cell cycle; schedule; colon adenocarcinoma cell lines

SN-38 is the active metabolite of CPT-11, a water-soluble derivative of the plant alkaloid camptothecin (CPT). Camptothecins exhibit a wide range of anti-neoplastic activity. They exert their cytotoxic effect through a single intracellular target, the nuclear enzyme topoisomerase I (topo I) (Hsiang et al, 1985; Hsiang and Liu, 1988), which relieves torsional strain introduced in the DNA duplex by active replication and transcription. The enzyme cleaves one of the strands of the duplex DNA, allowing the 5´-end of the cleaved strand to rotate around the internucleotide bond of the intact strand. Resealing of the cleaved strand after one or several strand passages completes enzyme action (Wang, 1985; Liu, 1989). CPT and its analogues slow the relegation step of the topo I catalytic cycle without affecting the DNA cleavage reaction. As a result, topo I-DNA adducts (cleavable complexes) are stabilized in the presence of CPT, resulting in single-strand DNA breaks.

Although the frequency of cleavable complexes correlates well with cytotoxicity of CPTs, the single-strand breaks are readily reversible upon removal of the drug and are not lethal to the cell. This suggests the involvement of additional factors. A model that accommodates the experimental data involves the collision of the replication machinery with the cleavable complex (Hsiang et al, 1989), causing the replication fork to arrest and a potentially lethal double-stranded break to occur, ultimately leading to apoptotic cell death.

This model explains the cytotoxicity of topo I-acting drugs towards cells in the S phase of the cell cycle. Therefore, although topo I is expressed throughout the cell cycle, it can be expected that topo I drugs will be phase-specific, and in clinical use schedule-dependent. Although camptothecin analogues are undergoing extensive investigation in clinical trials, relatively little information is available regarding the optimum schedule of these compounds. Therapeutic advantage with prolonged or repeated dosing schedules of topotecan and irinotecan in different mouse xenograft models has been described by several research groups (Kawato et al, 1991; Phillips et al, 1994; Houghton et al, 1995). The same results were obtained with topotecan in a soft-agar cloning system (Burris et al, 1992). In other mouse xenograft models CPT-11 appeared not to be markedly schedule-dependent in terms of toxicity, although the intermittent and prolonged schedules allowed moderately higher total dosages to be administered and were more active in this model (Bissery et al, 1996).

In addition to cytotoxic effects, CPTs cause cell cycle arrest in G2/M at low to moderate concentrations, probably as a result of failure to activate cdc2 kinase, an enzyme involved in phosphorlation reactions required to induce mitosis, whereas at higher concentrations arrest can also be observed in early S (Del Bino

*Received 22 February 1999*
*Revised 11 June 1999*
*Accepted 16 June 1999*

*Correspondence to*: RH te Poele

Current address:Signal Transduction & Molecular Pharmacology Team, CRC Centre for Cancer Therapeutics, The Institute of Cancer Research, E block, Sutton, Surrey SM2 5NE, UK

et al, 1990; Dubrez et al, 1995; Goldwasser et al, 1996). The arrest of cells in the G1 phase of the cell cycle by the topo I inhibitor TAN-1518 (Horiguchi et al, 1994), which has a distinct mechanism from CPT's, has been reported, and probably involves the tumour suppressor protein p53, known to regulate the G1/S checkpoint of the cell cycle and the onset of apoptosis following DNA damage.

In this study we have investigated: (i) what concentration, duration or exposure duration (ED; exposure × duration) of SN-38 treatment results in cytotoxic activity; (ii) how treatment with SN-38 affects cell cycle distribution and whether this influences the sensitivity of the cells; (iii) whether p53-induced cell cycle arrest may allow cells to escape apoptosis. Experiments were carried out with LS174T (wild-type p53) and HT29 (mutant non-functional p53) colon adenocarcinoma cell lines.

### MATERIALS AND METHODS

### Cell culture

HT29 (ATTC, Manassa, VA, USA) and LS174T (ATTC) colon adenocarcinoma cells were cultured in RPMI-1640 medium supplemented with 10% fetal bovine serum, penicillin 100 U ml$^{-1}$ and streptomycin 100 μg ml$^{-1}$ (Gibco-BRL, Paisley, UK). The cells were incubated at 37°C in a humidified atmosphere containing 5% carbon dioxide.

### SN-38

SN-38, a kind gift from Rhone Poulenc Rohrer, France, was dissolved in dimethylsulphoxide (DMSO; Sigma, Dorset, UK) and stock solutions were diluted in medium just before treatment. The final concentration of DMSO in the cell culture medium was 0.02% in all cases. The concentrations of SN-38 used for treatment in 3-(4,5-dimethylthiazal-2-yl)-2,5-diphenyltetrazolium bromide (MTT) and DNA analysis were 2, 5, 10, 20, 30, 50 and 100 ng ml$^{-1}$, and in those used in p53 and DNA double-staining for flow cytometry were 0, 6.25, 12.5, 25 and 50 ng ml$^{-1}$. The drug medium was replaced every 24 h. Detached cells were harvested and resuspended in the fresh drug medium.

### Immunoprecipitation and Western blotting

After exposure to SN-38 the cells were harvested by trypsinization, and washed three times with ice-cold phosphate-buffered saline (PBS). Cells that had become detached were harvested with the trypsinized cells and all were then incubated in lysis buffer for 30 min at 4°C (50 mM Tris–HCl (pH 7.4), 250 mM sodium chloride (NaCl) 5 mM EDTA, 50 mM NaF, 10 μg ml$^{-1}$ phenylmethylsulphonyl fluoride, 0.5 μg ml$^{-1}$ leupeptin, 2 μg ml$^{-1}$ soybean trypsin inhibitor I, 0.5 μg ml$^{-1}$ aprotine, 2 μg ml$^{-1}$ N-Tosyl-L-phenylanaline chloromethyl ketone, 0.1% Triton X-100) (Sigma). The lysate was centrifuged, and the supernatant was transferred to a fresh microfuge tube. The lysate was pre-cleared by overnight incubation with 100 μl of protein A/Sepharose (Pharmacia, St Albans, UK). Protein A/Sepharose was removed by centrifugation and the supernatant was transferred to a fresh microfuge tube. Monoclonal antibody to p53, either Pab 240 or Pab 1620, was added and incubated overnight. Then, 100 μl of protein A/Sepharose was added and incubated for another hour. The complex of protein A/Sepharose and p53 was washed three times with lysis buffer, after which 50 μl of Laemmli buffer (62.5 mM Tris–HCl pH 6.8, 10% (v/v) glycerol, 1% (w/v) sodium dodecyl sulphate, 5% (v/v) 2-mercaptoethanol, 0.0025% (w/v) bromophenol blue) (Sigma) was added.

For direct Western blots, without immunoprecipitation, washed cells were directly lysed in Laemmli buffer. The protein samples were boiled for 5 min and loaded on to a 7.5–15% acrylamide gel, with a 5% stacking gel. The gels were run for 4 h at 200 V, and were blotted on to polyvinyldifluoride (PVDF) membrane (Bio-Rad, Hemel Hempstead, UK) for 12–16 h at 20 V. Membranes were blocked in 5% blocking reagent (Boehringer Mannheim, Lewes, UK) in Tween–Tris-buffered saline (TTBS, 100 mM Tris–HCl pH 7.6, 0.9% (w/v) NaCl, 0.1% (v/v) Tween) for 1 h, and subsequently washed in TTBS (4 times 10 min). P53 and topo I blots were incubated with Pab 240 or anti-topo I (Pharmingen, San Diego, CA, USA). The washing step was repeated. Next the membranes were exposed to horseradish peroxidase rabbit anti-mouse (Dako, High Wycombe, UK). P53 and topo I bands were visualized by the enhanced chemiluminescence (ECL) detection system (Amersham, Little Chalfont, UK).

### Topoisomerase I activity assay

Nuclear extracts were prepared as described by Rubin et al (1994). Briefly, $5 \times 10^7$ cells were collected by trypsinization and washed in ice-cold PBS and nucleus buffer (NB; 2 mM $KH_2PO_4$, 5 mM magnesium chloride ($MgCl_2$), 150 mM NaCl, 1 mM EGTA, 0.1 mM dithiothreitol, 0.1 mM PMSF, pH 6.5). Cells were resuspended in 10 ml NB supplemented with 0.35% Triton X-100 and rotated for 10 min. After pelleting the nuclei they were washed in NB and resuspended in 1 ml NB containing 0.35 M NaCl and rotated for 2 h. The suspension was centrifuged at 17 000 ***g*** for 10 min, and 700 μl glycerol was added to the supernatant and stored at –20°C. Topoisomerase I activity in the nuclear extracts was determined using a DNA relaxation assay. The reaction mixture (final volume 20 μl) contained 0.25 μg supercoiled pHOT1 plasmid DNA (TopoGEN, Columbus, OH, USA), 10 mM Tris–HCl pH 7.5, 1 mM EDTA, 0.15 M NaCl, 0.1% bovine serum albumin 0.1 mM spermidine and 5% glycerol, to which a dilution of the nuclear extract was added. The mixture was incubated at 37°C for 2 h, after which 5 μl loading buffer (5% sarkosyl, 0.125% bromophenol blue, 25% glycerol) was added. The samples were loaded on a 1% agarose gel in TAE buffer (40 mM Tris–HCl, 20 mM glacial acetic acid, 10 mM EDTA) and were run in 4 × TAE buffer for 6 h at 40 V. After staining in water containing 0.5 μg ml$^{-1}$ ethidium bromide for 30 min and destaining in water for 30 min, the gels were photographed on Polaroid 665 film (Sigma).

### MTT assay

Cytotoxicity was determined by a modified MTT (Sigma) assay (Carmichael et al, 1987). Cells were seeded at 3000 cells per well in a 96-well microculture plate (Falcon, Becton-Dickinson, Oxford, UK). After 24-h incubation the medium was replaced with medium containing SN-38, and exposed for up to 7 days. The plates were developed by adding 50 μl MTT at 2 mg ml$^{-1}$ to each well, and incubated for a further 3 h. The plates were centrifuged at 450 ***g*** for 10 min, and the supernatant was carefully aspirated. The formazan crystals were dissolved in 200 μl of DMSO, and the absorbance at 570 nm was measured, with background correction

 © 1999 Cancer Research Campaign at 620 nm. Incubations were performed sixfold per test and each test was performed four times. The results were plotted against controls.

### DNA analysis

The DNA content of the cells was determined using a rapid one-step DNA staining technique, with the fluorescent dye propidium iodide, and apoptotic cells were discriminated by controlled extraction of low molecular weight DNA (Darzynkiewicz et al, 1994). Attached and detached cells were harvested and fixed overnight in 70% ethanol. The fixative was removed by centrifugation and the cells resuspended in 750 μl DNA extraction buffer, prepared by mixing 192 parts of 0.2 M $Na_2HPO_4$ with 8 parts of 0.1 M citric acid (pH 7.8). Following a 5-min incubation at room temperature the extraction buffer was removed by centrifugation and cells were resuspended in staining solution, 50 μg $ml^{-1}$ propidium iodide and 50 μg $ml^{-1}$ Ribonuclease A (Sigma). The cells were stained for 1 h in the dark at room temperature. Fluorescence was measured using a FACScan (Beckton-Dickinson) and results were analysed using Lysys II software (Beckton-Dickinson).

### P53 staining of cells for flow cytometry

The procedure was the same as for DNA analysis, but with additional steps between DNA extraction and DNA staining. The cells were washed in nucleus buffer (150 mM NaCl, 1 mM $KH_2PO_4$, 5 mM $MgCl_2$, 1 mM EGTA, 0.2 mM dithiothreitol, 1 mM PMSF (pH 6.4), and permeabilized for 20 min in nucleus buffer supplemented with 0.35% Triton-X (Sigma). After washing, the cells were incubated in primary antibody solution (DO7 1:10, Dako) for 1 h at room temperature. Primary antibody was removed by washing with PBS, after which secondary antibody was added (fluorescein isothiocyanate (FITC)-labelled anti-mouse, Sigma). The cells were incubated for 45 min at room temperature and washed with PBS. FITC and PI fluorescence were determined with the FACScan (Beckton-Dickinson). Cells were gated for each phase of the cell cycle according to the DNA profile and FITC fluorescence was determined in each gate. FITC fluorescence in SN-38-treated samples were expressed relative to control.

### Statistical analysis

Comparisons between treatments were made by analysis of variance (ANOVA), controlling for drug concentration and exposure duration. If the residuals from this analysis were not normally distributed, non-parametric ANOVA was used. Where ANOVA indicated differences between treatments, pair-wise comparisons were made using paired *t*-tests (normally distributed data) or Wilcoxon matched pairs signed ranks test (not normally distributed data).

### RESULTS

### P53 status, topoisomerase expression and activity

The p53 status of the HT29 and LS174T colon carcinoma cell lines was confirmed by immunoprecipitation experiments with the conformational antibodies Pab 240 and Pab 1620, which recognize the mutant and wild-type conformation of p53 respectively. As shown in Figure 1A only mutant conformation p53 was immunoprecipitated from HT29 cells, which confirms that the R273H mutant of p53 belongs to the class of structural mutants and is consistent with previous in vitro data (Rolley et al, 1995), whereas only wild-type conformation protein was precipitated from LS174T cells. Differences in topo I content or activity of both cell lines might influence cytotoxicity of SN-38 in these cell lines. However, the topo I content of both cell lines was similar by Western blot analysis of total cellular extract (Figure 1B). Topo I activity was also very similar in these cell lines, as demonstrated by the relaxation of supercoiled plasmid DNA (Figure 1C).

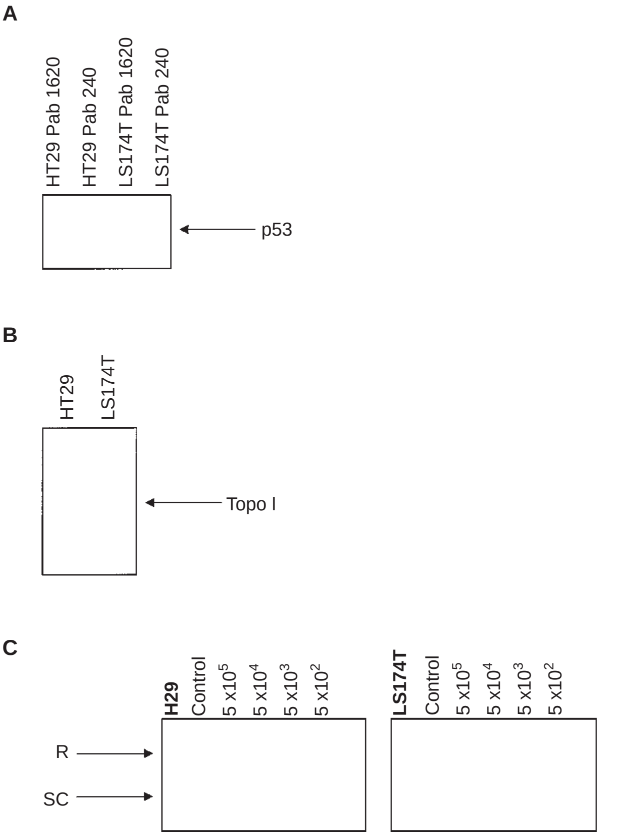

**Figure 1** P53 status and topo I levels and activity in HT29 and LS174T cells. (**A**) P53 western of Pab 240 and 1620 immunoprecipitated extracts, specific for mutant and wild-type p53 respectively, of $1 \times 10^6$ untreated cells. (**B**) Topoisomerase I Western blot of total cellular extract of $1 \times 10^5$ untreated cells. (**C**) Relaxation of supercoiled plasmid DNA by topo I from serial dilutions of nuclear extracts of untreated cells. Control lane contains only plasmid DNA. SC and R supercoiled and relaxed plasmid DNA

### Cell cycle effects of SN-38 in HT29 cells

Changes in cell cycle distribution of HT29 cells during exposure to SN-38 were both concentration- and duration-dependent ($P < 0.001$). The G1 phase of the cell cycle was rapidly emptied, within 24 h of exposure to concentrations ≥ 5 ng $ml^{-1}$ (Figure 2 A,B), with the fraction of cells in G1 remaining almost constant throughout the experiment at approximately 10%. Cells incubated with 2 ng $ml^{-1}$ SN-38 showed no significant change in G1 population. At 5 ng $ml^{-1}$ G1 was emptied until 10–20% of the cells were in this phase and remained at this percentage throughout the experiments.

*© 1999 Cancer Research Campaign* 

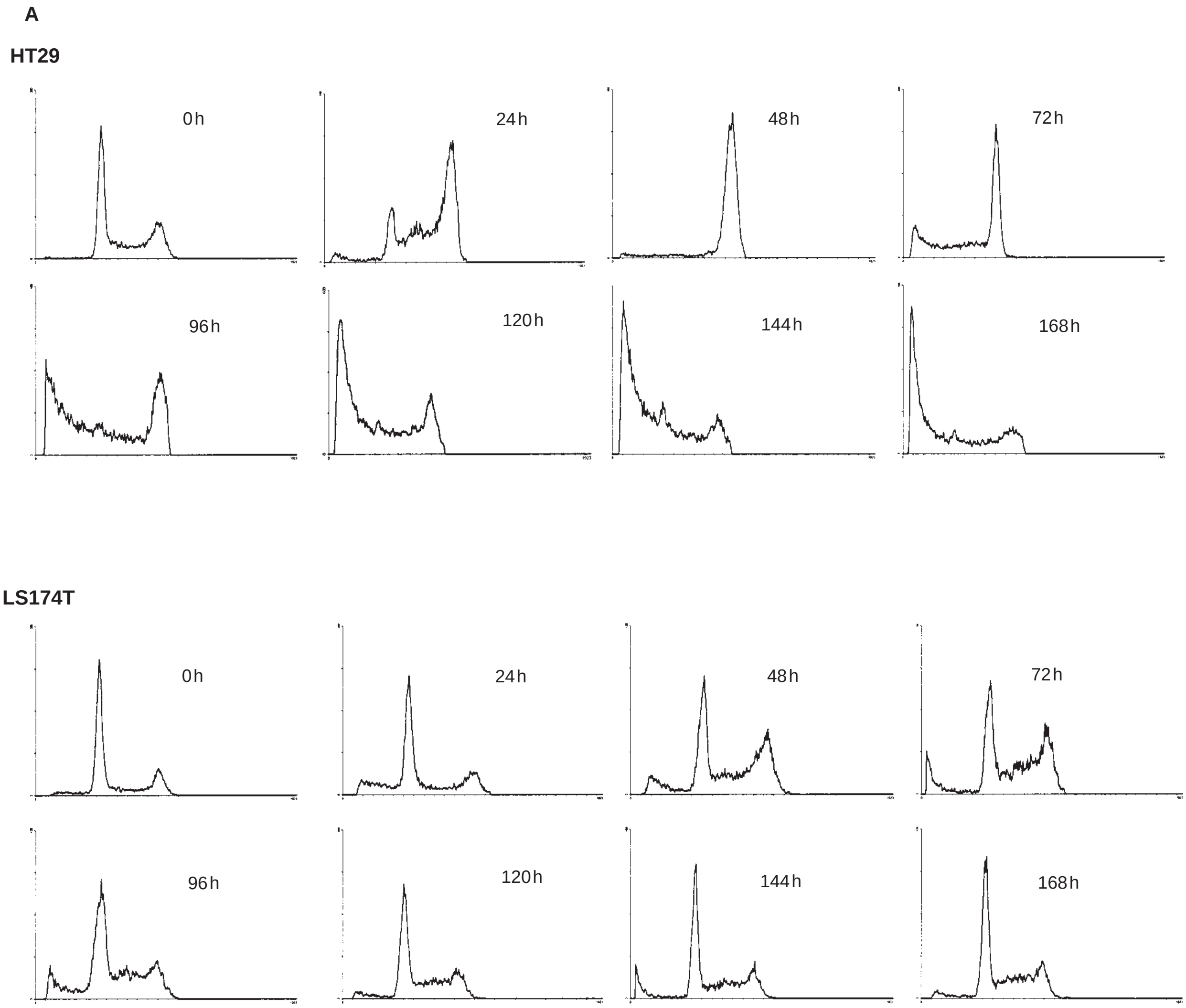

The S phase population of exponentially proliferating HT29 cells was 21.9 ± 8.3% (Figure 2B). After 24-h incubation there was a slight decrease in S phase occupancy in cells exposed to 5–30 ng ml$^{-1}$, which was much more apparent after 48 h at concentrations from 5 to 50 ng ml$^{-1}$. In contrast, at 100 ng ml$^{-1}$ a slight S phase accumulation could be seen at 24 h. After 48 h a repopulating of S phase occurred up to 72 h, after which a gradual decrease could be observed out to 168 h.

Cell cycle arrest in the G2/M phase of the cell cycle developed within 24 h of exposure to SN-38, and accumulation continued to a maximum at 48-h exposure (Figure 2 A,B). The maximum extent of accumulation in the G2/M phase was reached at concentrations of 10 and 20 ng ml$^{-1}$ (approximately 87 ± 3% of cells arrested in G2/M). At all concentrations which showed a G2/M block at 48 h, a rapid decrease in the G2/M population was apparent after 48 h (Figure 2 A,B).

The percentage of apoptotic cells as depicted in Figure 2B showed a significant increase with time and concentration ($P < 0.001$). The increase in the number of apoptotic cells became clear after 72 h, whereas up to 48 h there was little effect of SN-38 on apoptosis (Figure 2 A,B), except at the higher SN-38 concentrations. Apoptosis continued to increase at concentrations of ≥ 5 ng ml$^{-1}$ even out to 168 h. Dose effect reached plateau at 10–20 ng ml$^{-1}$ with exposures over 96 h, with little additional increase in apoptosis at higher concentrations (Figure 2B). Induction of apoptosis was confirmed by Western blotting for Poly ADP-ribose polymerase (PARP). The 85-kDa cleaved form of PARP could be observed 72 h after exposure to 25 ng ml$^{-1}$ SN-38 (results not shown).

### Cell cycle effects of SN-38 in LS174T cells

There was a strong concentration-dependent effect of SN-38 on the cell cycle distribution of LS174T cells ($P < 0.001$; Figure 2B), however, when accounting for variation with concentration there was no significant effect with exposure duration. At 2 ng ml$^{-1}$ the population in G1 consisted of about 35% of cells throughout the experiment. The G1 population decreased after 24-h exposure, with the effect being most marked at concentrations of 5–20 ng ml$^{-1}$ and a gradually smaller effect at higher concentrations up to 100 ng ml$^{-1}$ (Figure 2 A,B). After 24 h the G1 populations remained fairly constant at each concentration.

 *© 1999 Cancer Research Campaign*

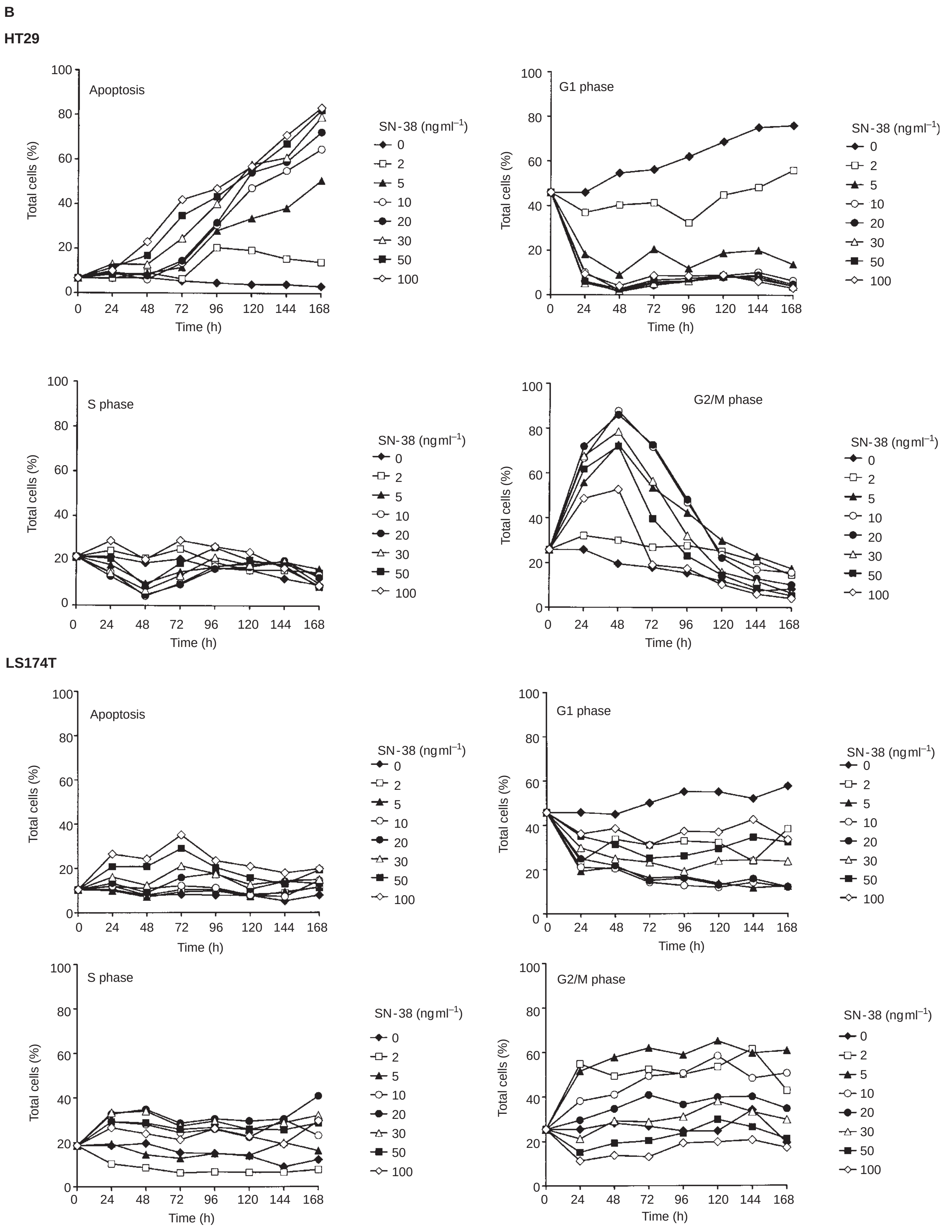

**Figure 2** Cell cycle analysis of HT29 and LS174T cells after exposure to SN-38. Time course of cell cycle dynamics by FACS analysis of $1 \times 10^4$ cells, extracted for low molecular weight DNA to discriminate apoptotic cells, stained with propidium iodide. (**A**) Typical DNA profiles for HT29 and LS174T cells exposed to 10 and 100 ng ml$^{-1}$ respectively. (**B**) Summary of the cell cycle data in HT29 and LS174T cells. Data are the averages of at least four experiments

*© 1999 Cancer Research Campaign*

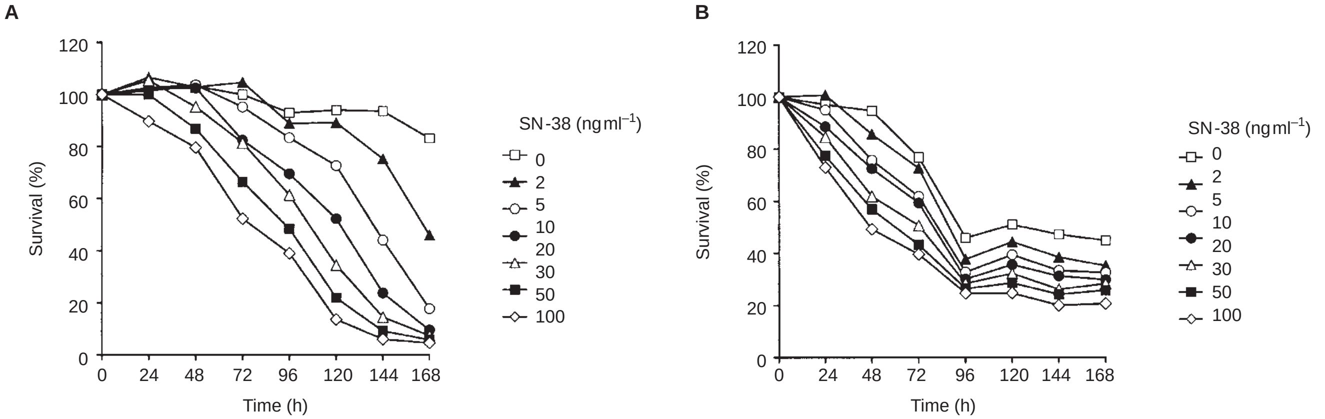

**Figure 3** Percentage survival of HT29 and LS174T cells after exposure to SN-38. Percentage survival was assessed by the MTT assay. (**A**) HT29, (**B**) LS174T. Data are the averages of at least four experiments

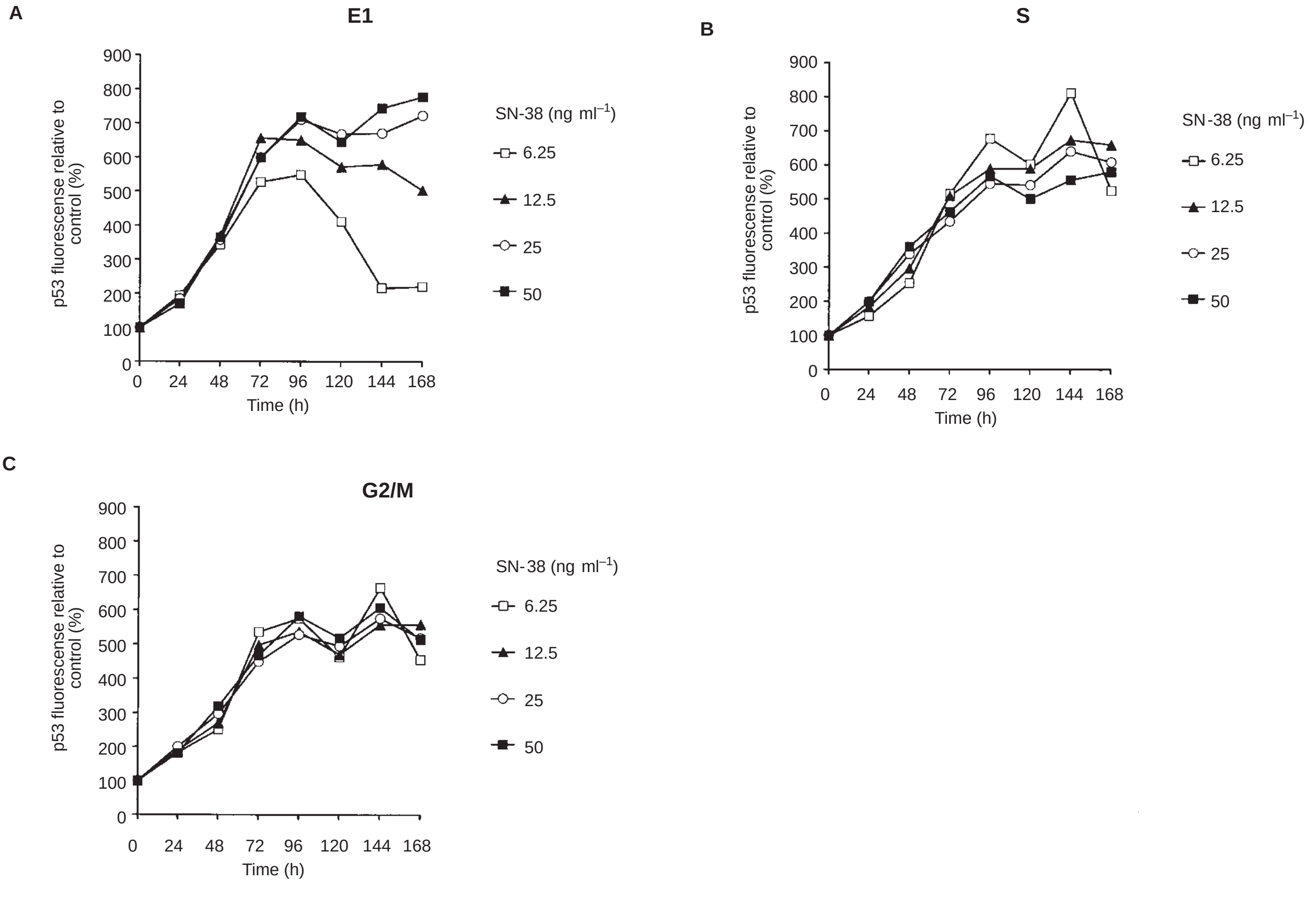

**Figure 4** P53 expression in the cell cycle phases of LS174T cells. P53 fluorescence relative to control in each phase of the cell cycle of LS174T cells gated out according to DNA profile. Data are the averages of four experiments

LS174T cells showed a concentration-dependent increase in the S phase population (Figure 2B), which was maximal at concentrations of 10–20 ng ml$^{-1}$ at approximately 35%, with no significant change with time after 24 h ($P > 0.05$).

G2/M arrest (Figure 2B) occurred within 24 h at concentrations of 2–10 ng ml$^{-1}$ and was maximal at concentrations of 2 and 5 ng ml$^{-1}$. There was little effect on the G2/M population at the higher concentrations, and again after 24 h there was no significant change in the G2/M population with time ($P > 0.05$).

A clear increase in apoptosis in LS174T cells (Figure 2B) was apparent only at the highest concentrations (50 and 100 ng ml$^{-1}$), for which apoptosis was maximal at 72 h. No PARP cleavage could be observed in LS174T cells exposed to 25 ng ml$^{-1}$ SN-38 (results not shown).

© 1999 Cancer Research Campaign

**Table 1** Schedule effect of SN-38 on apoptosis in HT29 and LS174T cells at the same ED (ED is exposure duration, exposure (ng $ml^{-1}$ multiplied by duration (h))

| A HT29<br>ED | Apoptosis ± SP<br>Schedule 1 | (T × C) | Schedule 2 | | Schedule 3 | |
|---|---|---|---|---|---|---|
| 240 | 9.9 (3.7–12.2) | 24 × 10 | 9.0 (7.0–10.2) | 48 × 5 | 15.6 (14.9–35.2)[b] | 120 × 2 |
| 480 | 9.3 ± 4.2 | 24 × 20 | 6.1 ± 2.0 | 48 × 10 | 28.0 ± 4.1[b,e] | 96 × 5 |
| 720 | 13.0 ± 8.8 | 24 × 30 | 12.3 ± 4.1 | 72 × 10 | 38.3 ± 5.1[b,e] | 144 × 5 |
| 960 | 7.9 ± 2.5 | 48 × 20 | 30.5 ± 4.2[e] | 96 × 10 | | |
| 1200 | 11.5 ± 6.5 | 24 × 50 | 47.2 ± 9.9[d] | 120 × 10 | | |
| 1440 | 12.8 ± 3.1 | 48 × 30 | 13.8 ± 3.3 | 72 × 20 | 54.9 ± 4.4[b,e] | 144 × 10 |
| 2400 | 10.0 ± 4.6 | 24 × 100 | 16.9 ± 4.4 | 48 × 50 | 54.1 ± 14.3[b,d] | 120 × 20 |
| 2880 | 40.0 ± 4.0 | 96 × 30 | 59.0 ± 6.7[d] | 144 × 20 | | |
| 3600 | 34.6 ± 4.6 | 72 × 50 | 57.7 ± 8.6[d] | 120 × 30 | | |
| 4800 | 23.0 ± 5.4 | 48 × 100 | 43.3 ± 2.8[d] | 96 × 50 | | |
| 7200 | 42.2 ± 2.0 | 72 × 100 | 67.1 ± 4.2[e] | 144 × 50 | | |

| B LS174T<br>ED | Schedule 1 | | Schedule 2 | | Schedule 3 | |
|---|---|---|---|---|---|---|
| 240 | 11.8 ± 1.6 | 24 × 10 | 7.0 ± 0.7[a] | 48 × 5 | 8.9 ± 3.4 1 | 20 × 2 |
| 480 | 13.2 ± 1.7 | 24 × 20 | 10.8 ± 2.9 | 48 × 10 | 9.8 ± 4.5 | 96 × 5 |
| 720 | 16.0 ± 1.1 | 24 × 30 | 12.1 ± 5.6 | 72 × 10 | 9.3 ± 2.0[d] | 144 × 5 |
| 960 | 9.0 ± 3.2 | 48 × 20 | 11.1 ± 6.4 | 96 × 10 | | |
| 1200 | 20.7 ± 0.9 | 24 × 50 | 8.2 ± 2.0[e] | 120 × 10 | | |
| 1440 | 12.3 ± 4.4 | 48 × 30 | 15.8 ± 3.3 | 72 × 20 | 7.1 ± 3.7[b] | 144 × 10 |
| 2400 | 26.4 ± 1.8 | 24 × 100 | 20.8 ± 12.3 | 48 × 50 | 19.0 ± 12.9[a] | 120 × 20 |
| 2880 | 20.8 ± 12.3 | 96 × 30 | 14.0 ± 6.3 | 144 × 20 | | |
| 3600 | 25.4 ± 9.3 | 72 × 50 | 12.8 ± 1.5 | 120 × 30 | | |
| 4800 | 24.2 ± 14.8 | 48 × 100 | 23.4 ± 13.5 | 96 × 50 | | |
| 7200 | 35.1 ± 10.4 | 72 × 100 | 12.9 ± 2.1[a,c] | 144 × 50 | | |

From schedule 1–3 increasing time and decreasing concentration.[a]Significant difference with schedule 1, [b]significant difference with schedule 2. [c]$P < 0.05$, [d]$P < 0.01$, [e]$P < 0.001$.

### Percentage survival after SN-38 exposure

Although there was little effect of SN-38 on apoptosis in LS174T cells, growth was inhibited, as can be seen in the decrease in percentage survival (Figure 3B). At 48 h growth was inhibited at all concentrations, and decreased with time and concentration up to 96-h exposure after which point the time effect decreased, probably because of fewer cycling cells in the control population.

In HT29 cells there was little effect of SN-38 on the percentage survival for the first 48 h, becoming apparent only after 48-h exposure. The decrease in the percentage survival decreased with time and concentration thereafter ($P < 0.001$), continuing out to 168 h. The minimum surviving population was 21% of control in LS174T cells, but only 5% in HT29 cells.

### P53 expression

The fact that LS174T cells were retained in G1, whereas HT29 cells rapidly progressed through G1 and S before arresting in G2/M, was an indication of the involvement of p53. To measure p53 expression in each compartment of the cell cycle, cells were dually stained for DNA and p53. As can be seen in Figure 4, p53 levels showed an approximate sevenfold increase relative to control on exposure to SN-38 in the G1, S and G2/M phases of the cell cycle. In each phase of the cell cycle p53 expression increased with time ($P < 0.001$), whereas only in G1 was there an effect of concentration on p53 expression ($P = 0.01$) with a concentration-dependent decrease in p53 levels after 96 h.

In HT29 cells there was no significant change in p53 levels ($P > 0.05$; results not shown). Western blot analyses of total cell extracts confirmed the results obtained by FACS analyses; with increased p53 levels in SN-38 (25 ng $ml^{-1}$) treated LS174T cells, but no change in HT29 cells (results not shown).

### Schedule effect of SN-38 at the same ED

The fact that there was a poor correlation between ED of SN-38 and apoptosis in both HT29 ($R^2 = 0.49$) and LS174T cells ($R^2 = 0.20$) indicated there might be a possible schedule effect. Therefore we analysed the extent of apoptosis of different schedules at the same ED (exposure × duration). In HT29 cells the more prolonged schedules at lower concentrations were significantly more active with respect to apoptosis than the shorter schedules at higher concentrations (Table 1A). In LS174T cells, however, there was a trend towards increased apoptosis in the shorter schedules at higher concentrations, although the difference reached statistical significance in a few cases only (Table 1B), possibly due to the lower level of apoptosis in this cell line.

### DISCUSSION

This study was designed to investigate the effect of concentration and exposure duration on the cytotoxicity of SN-38, with particular regard to cell cycle distribution and apoptosis in cells with different p53 status.

Although SN-38 is considered an S phase cytotoxic, it had very little effect on apoptosis and percentage survival in HT29 cells during the first 48 h. Exposure of HT29 cells to SN-38

© 1999 Cancer Research Campaign concentrations of 5 ng ml$^{-1}$ and higher resulted in a rapid emptying of the G1 phase of the cell cycle, and after going through S phase, cells accumulated in G2/M. Accumulation continued up to a maximum at 48 h. Cells damaged during replication in the S phase did not undergo apoptosis immediately and were allowed to traverse the S/G2 border before arresting in G2/M. S phase arrest at 48 h was relevant only at 100 ng ml$^{-1}$, indicating that extensive damage results in immediate arrest in this cell cycle phase. A dose-dependent S or G2/M arrest has also been reported in cell lines exposed to camptothecin, with cell cycle arrest in G2/M at low to moderate concentrations, whereas at higher concentrations arrest could also be observed in early S (Del Bino et al, 1990; Dubrez et al, 1995; Goldwasser et al, 1996).

In spite of continued drug exposure, HT29 cells were released from G2/M arrest, which resulted in a concomitant increase in apoptosis and a decrease in cell survival. Thereafter the S phase and G2/M phase depopulated with continued exposure, paralleled by an increase in apoptosis and a decrease in cell survival. We have observed the same pattern in ovarian cell lines with mutant or null p53 treated with SN-38 (RH te Poele, unpublished data), and similar results have been reported with 9-nitro and 9-amino camptothecin (Pantazis et al, 1993).

In LS174T cells apoptosis developed within 24 h of exposure at the higher concentrations and was maximal after 72-h exposure. Although the effect of SN-38 on apoptosis was modest, cell cycle arrest developed at all concentrations as reflected in the decrease in percentage survival compared to a control population. The cell cycle dynamics showed strong concentration dependence and little variation with exposure duration, suggesting that cells were frozen in the cell cycle, similar to the findings of Poot et al (1995) who reported accumulation of cells in each phase of the cell cycle with the parent compound camptothecin. At low concentrations LS174T cells progressed through S and accumulated in G2/M, and with increasing concentration arrested in the S phase, whereas at the highest concentration cells were retained in G1. Cells remained arrested for the duration of the exposure. In a continuation of these studies we found that, when SN-38 was withdrawn after 7 days, and LS174T cells were re-cultured in drug-free medium, cells remained arrested in the cell cycle. Moreover, cell lines with wild-type p53 responded in two ways to SN-38, either by undergoing apoptosis following transient G1 arrest, or by irreversible cell cycle arrest with phenotypical and biochemical characteristics of replicative senescence (RH te Poele, unpublished data).

In LS174T cells the response to SN-38 was faster, and was associated with increased expression of p53, and may involve more efficient recognition and an intact signal transduction pathway. This may also explain the increased sensitivity of LS174T cells to very low concentrations of SN-38 with respect to growth arrest. However, the fast response and increased sensitivity did not lead to an overall increase in apoptosis and decrease in cell survival as compared to HT29 cells. LS174T cells resisted apoptotic cell death by SN-38 through continued p53-associated cell cycle arrest, as opposed to the transient blockage in G2/M of HT29 cells, and suggests the possible involvement of p53 in prolonged cell cycle arrest in G1, S and G2/M. It has also been suggested that p53 is involved in prolonged G1 arrest in human fibroblasts after irradiation (Di Leonardo et al, 1994).

P53-associated G1 arrest or apoptosis indicates an additional cytotoxic mechanism of SN-38, because in G1 there is no DNA synthesis, and it has been shown that camptothecin-stabilized cleavable complexes are insufficient to stimulate increased p53 levels (Nelson and Kastan, 1994). The main genetic process in G1 is transcription and there is a possibility that interference in this process by SN-38 may generate a signal to p53. This is enforced by the observations that camptothecin causes rapid cessation of RNA synthesis (Bendixen et al, 1990), and was shown to have a cytotoxic mechanism that was not protected by aphidicolin (Goldwasser et al, 1996).

There was poor correlation between ED and apoptosis in both HT29 and LS174T cells indicating a possible schedule effect. More detailed investigation of the effect of different regimens at the same drug ED enforced this. Between the different schedules the regimens with prolonged exposure were consistently more effective in HT29 cells. An advantage with protracted schedules has been observed before in the laboratory and the clinical setting (Muggia et al, 1972; Kawato et al, 1991; Hochster et al, 1994; Houghton et al, 1995; Phillips et al, 1994; Slichenmyer et al, 1994). In LS174T cells, however, the shorter schedules at higher concentrations seemed to be more effective.

Although cells with intact p53 respond more efficiently to DNA damage than cells with mutated p53, the sensitivity to SN-38 seems mainly determined by the degree of cell cycle progression upon DNA damage. Cells, which do not progress through the cell cycle due to intact cell cycle check points, appear to have a reduced sensitivity to SN-38. A similar effect between cell cycle arrest and resistance to camptothecin has been described for leukaemic cell lines (Solary et al, 1996). We also found that p53 null or mutant ovarian cell lines showed transient arrest in S or G2/M followed by apoptosis with the protracted schedules. In tumour cells expressing wild-type p53 the situation is more complicated. Exposure to SN-38 either results in transient G1 arrest followed by apoptosis, in which case the protracted schedules are more active, or by undergoing permanent cell cycle arrest, when apoptosis could only be observed at the higher doses with short exposures (Figure 2B and RH te Poele, unpublished data), indicating the involvement of additional factors in determining the optimal schedule in this cell type. Although the differences between the two cell lines investigated are striking, and are at least in part due to their difference in p53 status, the influence of other differences between the cell lines can not be ruled out.

In view of the results presented here a pulsed schedule may prove more effective in LS174T cells, allowing cells to go back into cycle before cell cycle arrest becomes irreversible and an additional dose of drug is administered. This may also explain the observation from some clinical trials that repeated dose schedules were more effective (Fukuoka and Masuda, 1994; Von Hoff et al, 1994). The camptothecin derivatives topotecan or 9-amino-camptothecin might be more useful in this setting, because of their shorter plasma half-lives. However, considering that normal tissue contains wild-type p53, a high-dose pulsed schedule might also prove to be more toxic.

In contrast, continued, prolonged exposure in cells with mutant non-functional p53 appears to be more active. The minimum effective concentration in HT29 cells was 5–10 ng ml$^{-1}$, which is within the range of clinical relevance (Catimel et al, 1995; Canal et al, 1996). Above these concentrations, dose effect reached plateau with prolonged exposures. Prolonged exposure to SN-38, when there are differences in p53 status of the tumour and the normal tissue, might increase the therapeutic index of SN-38. These different activities of SN-38 schedules in p53 wild-type and mutant cells could also explain why some clinical trials

 © 1999 Cancer Research Campaign have not found a schedule effect (Armand, 1996), perhaps due to differences in p53 status in patient tumours.

The results of this study highlight the importance of cell cycle factors on the activity of topo I inhibitors. It can be anticipated that proteins involved in regulation of the cell cycle and modulation of apoptosis will play an important role in the clinical activity of these drugs. Additionally, the optimal treatment schedule for topo I inhibitors may be more dependent on the individual patient's tumour characteristics than the tumour site, suggesting a possible role for the use of molecular factors in determining treatment strategies.

### ACKNOWLEDGEMENTS

We would like to thank Dr Andrei Okorokov for the critical reading of the manuscript and helpful discussions. This work was supported by the Joint Research Board of St Bartholomew's Hospital.

### REFERENCES

Armand JP (1996) CPT-11: clinical experience in phase I studies. *Semin Oncol* **23**: 27–33

Bendixen C, Thomsen B, Alsner J and Westergaard O (1990) Camptothecin-stabilized topoisomerase I–DNA adducts cause premature termination of transcription. *Biochemistry* **29**: 5613–5619

Bissery MC, Vrignaud P, Lavelle F and Chabot GG (1996) Experimental antitumour activity and pharmacokinetics of the camptothecin analog irinotecan (CPT-11) in mice. *Anticancer Drugs* **7**: 437–460

Burris HAd, Hanauske AR, Johnson RK, Marshall MH, Kuhn JG, Hilsenbeck SG and Von Hoff DD (1992) Activity of topotecan, a new topoisomerase I inhibitor, against human tumor colony-forming units in vitro. *J Natl Cancer Inst* **84**: 1816–1820

Canal P, Gay C, Dezeuze A, Douillard JY, Bugat R, Brunet R, Adenis A, Herait P, Lokiec F and Mathieu-Boue A (1996) Pharmacokinetics and pharmacodynamics of irinotecan during a phase II clinical trial in colorectal cancer. Pharmacology and Molecular Mechanisms Group of the European Organization for Research and Treatment of Cancer. *J Clin Oncol* **14**: 2688–2695

Carmichael J, DeGraff WG, Gazdar AF, Minna JD and Mitchell JB (1987) Evaluation of a tetrazolium-based semiautomated colorimetric assay: assessment of radiosensitivity. *Cancer Res* **47**: 943–946

Catimel G, Chabot GG, Guastalla JP, Dumortier A, Cote C, Engel C, Gouyette A, Mathieu-Boue A, Mahjoubi M and Clavel M (1995) Phase I and pharmacokinetic study of irinotecan (CPT-11) administered daily for three consecutive days every three weeks in patients with advanced solid tumors [see comments]. *Ann Oncol* **6**: 133–140

Darzynkiewicz Z, Li X and Gong J (1994) Assays of cell viability: discrimination of cells dying by apoptosis. *Methods Cell Biol* **41**: 15–38

Del Bino G, Skierski JS and Darzynkiewicz Z (1990) Diverse effects of camptothecin, an inhibitor of topoisomerase I, on the cell cycle of lymphocytic (L1210, MOLT-4) and myelogenous (HL-60, KG1) leukemic cells. *Cancer Res* **50**: 5746–5750

Di Leonardo A, Linke SP, Clarkin K and Wahl GM (1994) DNA damage triggers a prolonged p53-dependent G1 arrest and long-term induction of Cip1 in normal human fibroblasts. *Genes Dev* **8**: 2540–2551

Dubrez L, Goldwasser F, Genne P, Pommier Y and Solary E (1995) The role of cell cycle regulation and apoptosis triggering in determining the sensitivity of leukemic cells to topoisomerase I and II inhibitors. *Leukemia* **9**: 1013–1024

Fukuoka M and Masuda N (1994) Clinical studies of irinotecan alone and in combination with cisplatin. *Cancer Chemother Pharmacol* **34**: S105–S111

Goldwasser F, Shimizu T, Jackman J, Hoki Y, O'Connor PM, Kohn KW and Pommier Y (1996) Correlations between S and G2 arrest and the cytotoxicity of camptothecin in human colon carcinoma cells. *Cancer Res* **56**: 4430–4437

Hochster H, Liebes L, Speyer J, Sorich J, Taubes B, Oratz R, Wernz J, Chachoua A, Raphael B, Vinci RZ and et al (1994) Phase I trial of low-dose continuous topotecan infusion in patients with cancer: an active and well-tolerated regimen. *J Clin Oncol* **12**: 553–559

Horiguchi T, Hayashi K, Tsubotani S, Iinuma S, Harada S and Tanida S (1994) New naphthacenecarboxamide antibiotics, TAN-1518 A and B, have inhibitory activity against mammalian DNA topoisomerase I. *J Antibiotics* **47**: 545–556

Houghton PJ, Cheshire PJ, Hallman JDn, Lutz L, Friedman HS, Danks MK and Houghton JA (1995) Efficacy of topoisomerase I inhibitors, topotecan and irinotecan, administered at low dose levels in protracted schedules to mice bearing xenografts of human tumors. *Cancer Chemother Pharmacol* **36**: 393–403

Hsiang YH and Liu LF (1988) Identification of mammalian DNA topoisomerase I as an intracellular target of the anticancer drug camptothecin. *Cancer Res* **48**: 1722–1726

Hsiang YH, Hertzberg R, Hecht S and Liu LF (1985) Camptothecin induces protein-linked DNA breaks via mammalian DNA topoisomerase I. *J Biol Chem* **260**: 14873–14878

Hsiang YH, Lihou MG and Liu LF (1989) Arrest of replication forks by drug-stabilized topoisomerase I-DNA cleavable complexes as a mechanism of cell killing by camptothecin. *Cancer Res* **49**: 5077–5082

Kawato Y, Furuta T, Aonuma M, Yasuoka M, Yokokura T and Matsumoto K (1991) Antitumor activity of a camptothecin derivative, CPT-11, against human tumor xenografts in nude mice. *Cancer Chemother Pharmacol* **28**: 192–198

Liu LF (1989) DNA topoisomerase poisons as antitumor drugs. [Review]. *Annu Rev Biochem* **58**: 351–375

Muggia FM, Creaven PJ, Hansen HH, Cohen MH and Selawry OS (1972) Phase I clinical trial of weekly and daily treatment with camptothecin (NSC-100880): correlation with preclinical studies. *Cancer Chemotherapy Reports – Part 1* **56**: 515–521

Nelson WG and Kastan MB (1994) DNA strand breaks: the DNA template alterations that trigger p53-dependent DNA damage response pathways. *Mol Cell Biol* **14**: 1815–1823

Pantazis P, Mendoza JT, Early JA, Kozielski AJ, Natelson EA and Giovanella BC (1993) 9-Nitro-camptothecin delays growth of U-937 leukemia tumors in nude mice and is cytotoxic or cytostatic for human myelomonocytic leukemia lines in vitro. *Eur J Haematol* **50**: 81–89

Phillips PC, Janss A and Kaufmann SH (1994) Topoisomerase I inhibitor schedule dependent activity and determinants of cytotoxicity in human brain tumors cell lines. *Proc Annu Meet Am Assoc Cancer Res* **35**

Poot M, Hiller KH, Heimpel S and Hoehn H (1995) Distinct patterns of cell cycle disturbance elicited by compounds interfering with DNA topoisomerase I and II activity. *Exp Cell Res* **218**: 326–330

Rolley N, Butcher S and Milner J (1995) Specific DNA binding by different classes of human p53 mutants. *Oncogene* **11**: 763–770

Rubin E, Pantazis P, Bharti A, Toppmeyer D, Giovanella B and Kufe D (1994) Identification of a mutant human topoisomerase I with intact catalytic activity and resistance to 9-nitro-camptothecin. *J Biol Chem* **269**: 2433–2439

Slichenmyer WJ, Rowinsky EK, Grochow LB, Kaufman SH and Donehauwer RC (1994) Camptothecin analogues: studies from The Johns Hopkins Oncology Center. *Cancer Chemother Pharmacol* **34**: S53–S57

Solary E, Dubrez L, Eymin B, Bertrand R and Pommier Y (1996) [Apoptosis of human leukemic cells induced by topoisomerase I and II inhibitors]. *Bull Cancer (Paris)* **83**: 205–212

Von Hoff D, Burris HR, Eckardt J, Rothenberg M, Fields SM, Chen SF and Kuhn JG (1994) Preclinical and phase I trials of topoisomerase I inhibitors. *Cancer Chemother Pharmacol*

Wang JC (1985) DNA topoisomerases. *Annu Rev Biochem* **54**: 665–697

© 1999 Cancer Research Campaign 
